# Critical Illness, Major Surgery, and Other Hospitalizations and Active and Disabled Life Expectancy

**DOI:** 10.1001/jamanetworkopen.2025.4208

**Published:** 2025-04-03

**Authors:** Thomas M. Gill, Emma X. Zang, Linda Leo-Summers, Evelyne A. Gahbauer, Robert D. Becher, Lauren E. Ferrante, Ling Han

**Affiliations:** 1 Department of Internal Medicine, Yale School of Medicine, New Haven, Connecticut; 2 Department of Sociology, Yale University, New Haven, Connecticut; 3 Department of Surgery, Yale School of Medicine, New Haven, Connecticut

## Abstract

**Importance:**

Estimates of active and disabled life expectancy, defined as the projected number of remaining years without and with disability in essential activities of daily living, are commonly used by policymakers to forecast the functional well-being of older persons.

**Objective:**

To determine how estimates of active and disabled life expectancy differ based on exposure to intervening illnesses and injuries (or events).

**Design, Setting, and Participants:**

This prospective cohort study was conducted in south-central Connecticut from March 1998 to December 2021 among 754 community-living persons aged 70 years or older who were not disabled. Data were analyzed from January 25 to September 18, 2024.

**Exposures:**

Exposure to intervening events, which included critical illness, major elective and nonelective surgical procedures, and hospitalization for other reasons, was assessed each month.

**Main Outcomes and Measures:**

Disability in 4 essential activities of daily living (bathing, dressing, walking, and transferring) was ascertained each month. Active and disabled life expectancy were estimated using multistate life tables under a discrete-time Markov process assumption.

**Results:**

The study included 754 community-living older persons who were not disabled (mean [SD] age, 78.4 [5.3] years; 487 female [64.6%]; 67 Black [8.9%], 4 Hispanic [0.5%], 682 non-Hispanic White [90.5%], and 1 other race [0.1%]). Within 5-year age increments from 70 to 90 years, active life expectancy decreased monotonically as the number of admissions for critical illness and other hospitalization increased. For example, at age 70 years, sex-adjusted active life expectancy decreased from 14.6 years (95% CI, 13.9-15.2 years) in the absence of a critical illness admission to 11.3 years (95% CI, 10.3-12.2 years), 8.1 years (95% CI, 6.3-9.9 years), and 4.0 years (95% CI, 2.6-5.7 years) in the setting of 1, 2, or 3 or more critical illness admissions, respectively. Corresponding values for other hospitalization were 19.4 years (95% CI, 18.0-20.8 years), 13.5 years (95% CI, 12.2-14.7 years), 10.0 years (95% CI, 8.9-11.2 years), and 7.0 years (95% CI, 6.1-7.9 years), respectively. Consistent monotonic reductions were observed for sex-adjusted estimates in active life expectancy for nonelective but not elective surgical procedures as the number of admissions increased; for example, at age 70 years, estimates of active life expectancy were 13.9 years (95% CI, 13.3-14.5 years), 11.7 years (95% CI, 10.5-12.8 years), and 9.2 years (95% CI, 7.4-11.0 years) for 0, 1, and 2 or more nonelective surgical admissions, respectively; corresponding values were 13.4 years (95% CI, 12.8-3-14.1 years), 14.6 years (95% CI, 13.5-15.5 years), and 12.6 years (95% CI, 11.5-13.8 years) for elective surgical admissions. Sex-adjusted disabled life expectancy decreased as the number of admissions increased for critical illness and other hospitalization but not for nonelective or elective surgical procedures; for example, at age 70 years, disabled life expectancy decreased from 4.4 years (95% CI, 3.5-5.8 years) in the absence of other hospitalization to 3.4 years (95% CI, 2.8-4.1 years), 3.4 years (95% CI, 2.7-4.2 years), and 2.3 years (95% CI, 1.9-2.8 years) in the setting of 1, 2, or 3 or more other hospitalizations, respectively.

**Conclusions and Relevance:**

This study found that active life expectancy among community-living older persons who were not disabled was considerably diminished in the setting of serious intervening illnesses and injuries. These findings suggest that prevention and more aggressive management of these events, together with restorative interventions, may be associated with improved functional well-being among older persons.

## Key Points

**Question:**

How do estimates of active and disabled life expectancy differ based on exposure to intervening illnesses and injuries?

**Findings:**

Among 754 community-living older persons who were not disabled and who were assessed monthly for more than 23 years in this cohort study, active life expectancy decreased monotonically as the number of hospital admissions increased for critical illness, major nonelective surgical procedures, and reasons other than critical illness or major surgical procedures but not for major elective surgical procedures.

**Meaning:**

These findings suggest that prevention and more aggressive management of serious intervening illnesses and injuries, together with restorative interventions, may be associated with improved functional well-being among older persons.

## Teaser

This cohort study estimates active and disabled life expectancy by exposure to critical illness, major surgical procedure, and other hospitalization events among older individuals.

## Introduction

Maintaining independent function is the number one goal of most older persons.^1^ Activities essential for maintaining independence include bathing, dressing, walking, and transferring from a chair. Estimates of active and disabled life expectancy, defined as the projected number of remaining years without and with disability in these activities of daily living, are commonly used by policymakers to forecast the functional well-being of older persons.^2,3,4,5^ Differences in active and disabled life expectancy have primarily been reported by age and sex,^3,5,6^ although some studies have evaluated differences by other factors, such as education, physical activity, multimorbidity, and individual diseases.^7,8,9,10^ However, prior research has shown that disability in older persons arises most commonly in the setting of intervening illnesses and injuries and that the contributions of these events to the onset and progression of disability far exceed those of individual risk factors.^11,12^ Nonetheless, the role of these intervening events in active and disabled life expectancy has not been previously evaluated to our knowledge.

The objective of this study was to determine how estimates of active and disabled life expectancy differ based on exposure to intervening illnesses and injuries. We focused on critical illness, major surgical procedure, and hospitalization for other reasons given their strong associations with disability and functional decline.^12,13,14^ To accomplish our objective, we used high-quality data from a unique longitudinal study of community-living older persons that includes monthly assessments of functional status and complete ascertainment of intervening events for more than 23 years. Because many intervening illnesses and injuries are amenable to prevention or to more aggressive management and restorative interventions, results of this study may inform strategies and policies to improve the functional well-being of older persons.

## Methods

### Study Population

Participants in this cohort study included 754 community-living persons aged 70 years or older who were not disabled in any of 4 essential activities of daily living: bathing, dressing, walking inside the home, and transferring from a chair. The assembly of the cohort, which took place between March 1998 and October 1999, has been described in detail elsewhere.^15,16^ In brief, potential participants were identified from a computerized list of 3157 age-eligible members of a large health plan in south-central Connecticut. Eligibility was determined during a screening telephone interview and was confirmed during an in-home assessment. Among 2753 members who were alive and could be contacted, 4.6% refused to complete the screening interview, and 75.2% of eligible members agreed to participate. The 248 persons who declined to participate did not differ from those who were enrolled in terms of age or sex. The study protocol was approved by the Human Investigation Committee at Yale University, and all participants provided informed consent. We followed the Strengthening the Reporting of Observational Studies in Epidemiology (STROBE) reporting guideline.

### Data Collection

Assessments were completed by trained nurse researchers at baseline, while telephone interviews were completed each month by a separate team of researchers through December 2021. For participants who had significant cognitive impairment or were otherwise unavailable, a proxy informant was interviewed using a rigorous protocol.^17^ Deaths were ascertained by review of local obituaries, from an informant during a subsequent interview, or by both methods. A total of 727 participants (96.4%) died after a median of 111 months, while 43 participants (5.7%) withdrew from the study after a median of 27 months. Data were otherwise available for 99.2% of 87 164 monthly interviews. The cohort has been linked to Medicare data.^18^

#### Descriptive Characteristics

During the baseline assessment, data were collected on demographic characteristics, cognitive impairment,^19^ and 9 self-reported, physician-diagnosed chronic conditions. Participants were asked to self-identify their race, which was assessed primarily for descriptive purposes. There was a single question in the baseline assessment that asked about race, with 4 response items: Black, Hispanic, White, and other.

#### Ascertainment of Intervening Illnesses or Injuries

Intervening illnesses or injuries, referred to interchangeably as* intervening events*, included hospitalizations with an intensive care unit (ICU) admission (hereafter, *critical illness*), with major surgical procedure, or for other reasons (ie, not critical illness or major surgical procedure). The primary source of information on hospitalizations was linked Medicare claims data, which were available for nearly all admissions.^20^ Information on some hospitalizations (ie, those without a Medicare record) was obtained during monthly interviews.^21^ Participants were asked whether they had stayed at least overnight in a hospital since the last interview. To ensure complete ascertainment of intervening events, careful medical record review was completed for all individuals who died.

Hospitalizations were subsequently classified as critical illness, major surgical procedure, or other using methods that have been previously described.^13,14^ For fee-for-service Medicare, an ICU admission was defined as any critical care revenue code, excluding psychiatric or intermediate critical care. For managed Medicare and hospitalizations ascertained from monthly interviews, ICU admissions were identified through review of corresponding medical records.^8^ To identify participants who had undergone an operation, Medicare files and monthly interview data on self-reported surgical procedures, verified by medical record review, were used.^13^ Major surgical procedure was defined as any procedure in an operating room requiring the use of general anesthesia for a nonpercutaneous, nonendoscopic, invasive operation.^22^ Major surgical procedures identified from Medicare files were classified as elective or nonelective by an indicator variable; nonelective surgical procedures included urgent and emergent operations.^23^ Major surgical procedures identified by self-report and medical record review were classified as nonelective or elective based on the timing of surgical procedure.^23^ Hospitalizations that did not meet criteria for critical illness or major surgical procedure were classified as *other*. For descriptive purposes, we categorized reasons for critical illness admissions and other hospitalizations and the types of major surgical procedure, as described in the eMethods in Supplement 1.

#### Assessment of Disability

Complete details regarding the assessment of disability, including reliability and accuracy, are provided elsewhere.^17,24^ During the monthly interviews, participants were asked, “At the present time, do you need help from another person to [complete the task]?” for each of 4 activities of daily living that were assessed during the screening telephone interview.^17^ Participants who needed help with any activity were considered to be disabled. Conversely, participants who did not need help were considered to be not disabled (or independent). To address the small amount of missing data on disability, multiple imputation was used with 100 random draws per missing observation.^25^

### Statistical Analysis

Baseline characteristics were described as mean and SD for continuous variables and frequency and percentage for categorical variables. Exposures to critical illness, major surgical procedure, and hospitalization for other reasons were each summarized as the total number of events per 1000 person-months.

Active and disabled life expectancy were estimated using multistate life tables under a discrete-time Markov process assumption.^26^ Each participant’s monthly probabilities of transitioning from their current state (active or disabled) to 1 of 3 states (active, disabled, or dead) were determined using a multinomial logistic regression model, with death as an absorbing state and age in months as an explanatory variable. Values for active and disabled life expectancy were estimated separately based on time-varying exposure to critical illness, major surgical procedure, and other hospitalization and are reported by age in 5-year increments from 70 to 90 years. Exposure to each type of intervening event over the entire follow-up period was categorized as a cumulative count of 0, 1, 2, or 3 or more admissions. For each age- and exposure-specific set of estimates, mean values and 95% CIs were calculated using 1000 bootstrap samples.^26,27^

For each type of intervening event, primary models adjusted for sex, although models were also run separately for female and male participants. Separate models were also run for elective and nonelective major surgical procedure, but the exposure had to be categorized as 0, 1, or 2 or more admissions because the original models did not converge owing to small cell sizes for 3 or more admissions. Months that also included critical illness or major surgical procedure were classified as not exposed for other hospitalization based on prior research indicating that disabling effects of these 2 types of intervening events are more pronounced than those of other hospitalization.^12^ To address potential confounding from this hierarchical approach, models for other hospitalization were adjusted for time-varying exposure to critical illness or major surgical procedure, in addition to sex. Models for critical illness and major surgical procedure, respectively, were not adjusted for other hospitalizations that occurred in the same month; this was done for 2 reasons. First, confounding would be unlikely because of the previously demonstrated hierarchy.^12^ Second, the co-occurrence of these events and other hospitalizations in the same month was low (16.8% for critical illness and 8.7% for major surgical procedure). Finally, for completeness, values for total life expectancy (the sum of active and disabled life expectancy) were calculated for results on critical illness, major surgical procedure, and other hospitalization.

We used SAS statistical software version 9.4 (SAS Institute) for descriptive analyses, Stata statistical software version 16 (StataCorp) for multinomial logistic regression analyses, and customized R code in the RStudio environment (RStudio software version 4.2.3 [RStudio]) for bootstrapped estimates. Data were analyzed from January 25 to September 18, 2024.

## Results

Among 754 participants (mean [SD] age, 78.4 [5.3] years; 487 female [64.6%]; 67 Black [8.9%], 4 Hispanic [0.5%], 682 non-Hispanic White [90.5%], and 1 other race [0.1%]), 298 individuals (39.5%) lived alone and 86 individuals (11.4%) were cognitively impaired. Participants had a mean (SD) of 12.0 (2.9) years of education and 1.8 (1.2) chronic conditions.

Based on a median (IQR) follow-up of 111 (55-164) months, exposure rates per 1000 person-months for critical illness, major surgical procedure, and other hospitalization were 5.6 events (95% CI, 5.0-6.2 events), 7.1 events (95% CI, 6.5-7.8 events), and 30.6 events (95% CI, 28.7-32.8 events), respectively. Reasons and types of these intervening events are provided in eTables 1, 2, and 3 in Supplement 1. Mean estimates for active and disabled life expectancy anchored at age 70 years were 13.5 years (95% CI, 12.9-14.0 years) and 2.9 years (95% CI, 2.6-3.1 years), respectively. Corresponding values were 13.4 years (95% CI, 12.7-14.0 years) and 3.5 years (95% CI, 3.2-3.8 years) for females and 13.7 years (95% CI, 12.6-14.6 years) and 1.8 years (95% CI, 1.5-2.0 years) for males.

**Figure 1.**
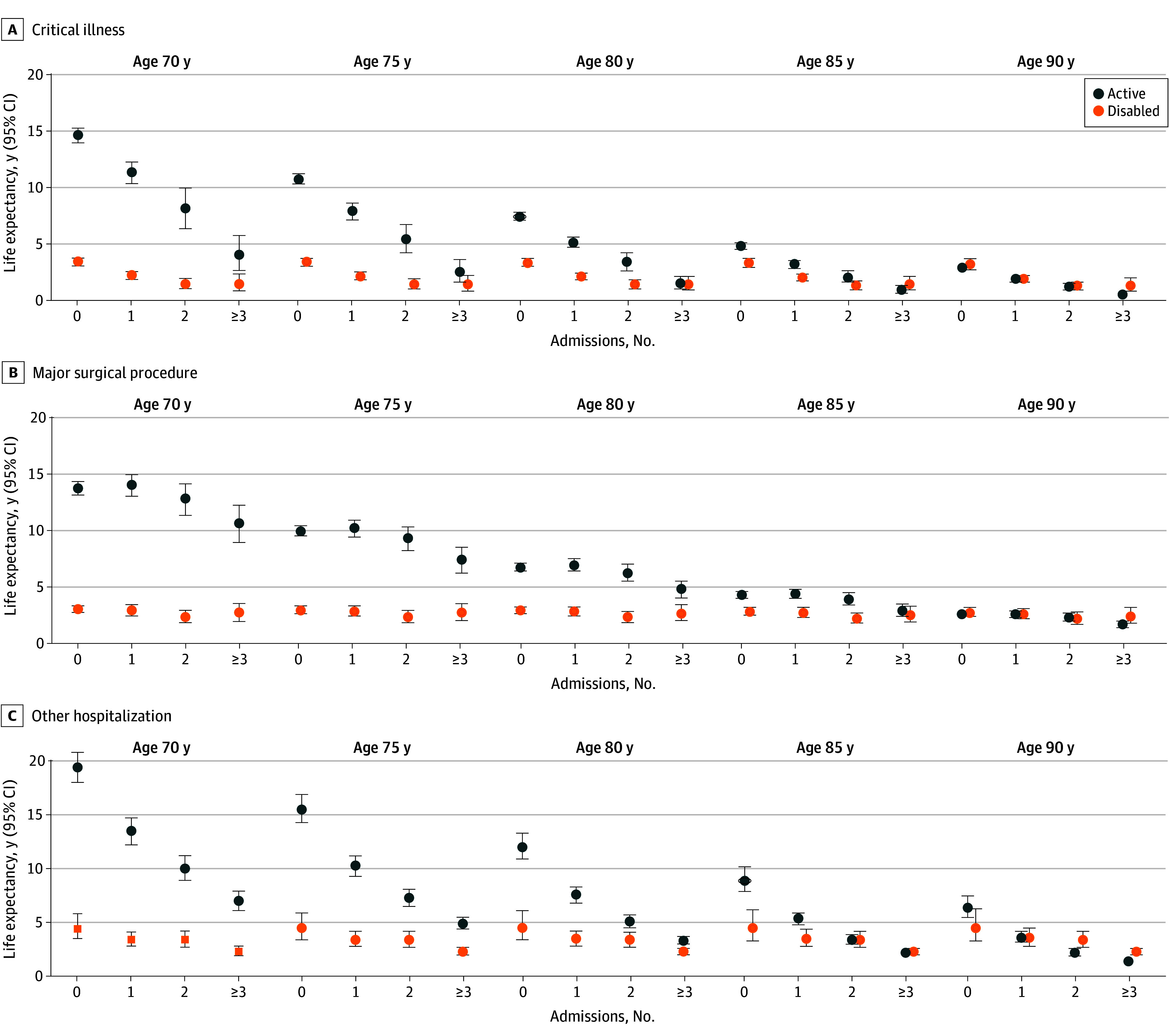
Life Expectancy by Age and Critical Illness, Major Surgical Procedure, and Other Hospitalization Admissions For critical illness and major surgical procedure, values are adjusted for sex. For other hospitalization, values are adjusted for sex and time-varying exposure to critical illness or major surgical procedure.

Figure 1 provides estimates for active and disabled life expectancy by age and number of admissions for critical illness, major surgical procedure, and other hospitalization. Exact values are provided in eTable 4 in Supplement 1. For each age group, active life expectancy decreased monotonically as the number of admissions for critical illness and other hospitalization increased. For example, at age 70 years, active life expectancy decreased from 14.6 years (95% CI, 13.9-15.2 years) in the absence of a critical illness admission to 11.3 years (95% CI, 10.3-12.2 years), 8.1 years (95% CI, 6.3-9.9 years), and 4.0 (95% CI, 2.6-5.7 years) years in the setting of 1, 2, or 3 or more critical illness admissions, respectively. Corresponding values for other hospitalization were 19.4 years (95% CI, 18.0-20.8 years), 13.5 years (95% CI, 12.2-14.7 years), 10.0 years (95% CI, 8.9-11.2 years), and 7.0 years (95% CI, 6.1-7.9 years), respectively. Disabled life expectancy also decreased as the number of admissions for critical illness and other hospitalization increased, but these reductions were not consistently monotonic. For example, at age 70 years, disabled life expectancy decreased from 4.4 years (95% CI, 3.5-5.8 years) in the absence of other hospitalization to 3.4 years (95% CI, 2.8-4.1 years), 3.4 years (95% CI, 2.7-4.2 years), and 2.3 years (95% CI, 1.9-2.8 years) in the setting of 1, 2, or 3 or more other hospitalizations, respectively. Corresponding values for total life expectancy are provided in eTable 5 in Supplement 1.

**Figure 2.**
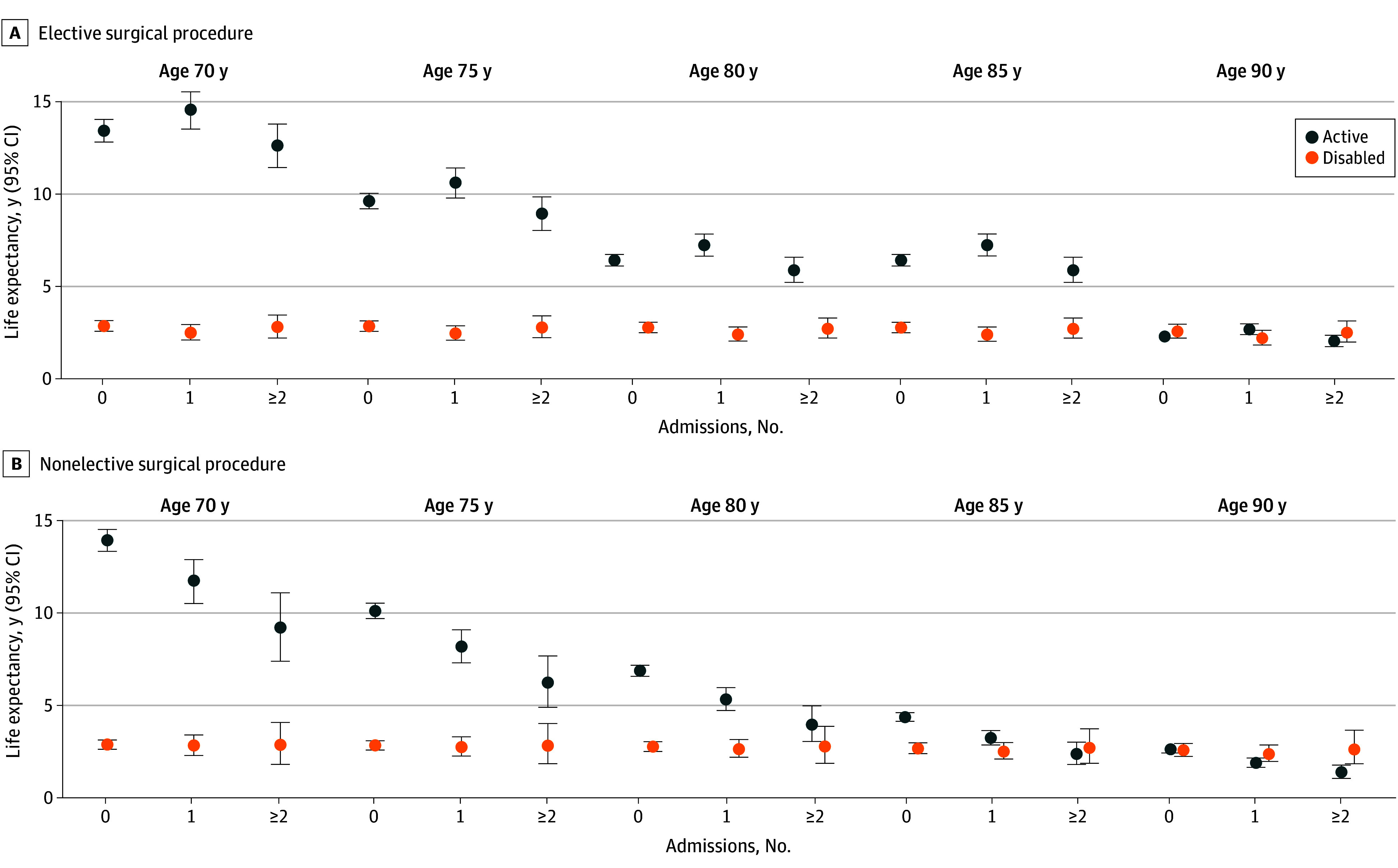
Life Expectancy by Age and Elective and Nonelective Major Surgical Admissions Values are adjusted for sex. The exposure had to be categorized into 3 groups because models with 4 groups did not converge owing to small cell sizes for 3 or more admissions.

For major surgical procedure, active life expectancy tended to decrease for each age group as the number of admissions increased, although this was not consistent and the magnitude of these reductions was much smaller than those associated with critical illness and other hospitalization. Corresponding values for disabled life expectancy changed relatively little as the number of admissions increased. As shown in Figure 2 and eTable 6 in Supplement 1, results differed substantially between elective and nonelective surgical procedures, with consistent monotonic reductions in active life expectancy observed for the latter but not the former as the number of admissions increased. For example, at age 70 years, estimates of active life expectancy were 13.9 years (95% CI, 13.3-14.5 years), 11.7 years (95% CI, 10.5-12.8 years), and 9.2 years (95% CI, 7.4-11.0 years) for 0, 1, and 2 or more nonelective surgical admissions, respectively; corresponding values were 13.4 years (95% CI, 12.8-3-14.1 years), 14.6 years (95% CI, 13.5-15.5 years), and 12.6 years (95% CI, 11.5-13.8 years), respectively, for elective surgical admissions. For elective and nonelective surgical procedures, corresponding estimates for disabled life expectancy changed relatively little as the number of admissions increased.

Sex-specific results were consistent with overall results for all 3 intervening event types, as shown for critical illness in Table 1, major surgical procedure in Table 2, and other hospitalization in Table 3. Furthermore, for each subgroup defined by age and number of admissions, all estimates of disabled life expectancy were larger in female than male participants, while sex-specific differences in active life expectancy were less striking and more variable, with 1 exception. For 3 or more admissions for major surgical procedure, values were consistently much larger in male than female participants. Corresponding values for total life expectancy are provided in eTable 7 in Supplement 1.

**Table 1.** Life Expectancy According to Age and Critical Illness Admissions by Sex

Age, y	Life expectancy, mean (95% CI), y				
	Females		Males		
	Active	Disabled	Active	Disabled	
70					
0 Admissions	14.5 (13.8-15.2)	4.1 (3.7-4.7)	14.5 (13.3-15.6)		2.2 (1.8-2.8)
1 Admissions	10.3 (9.0-11.5)	2.5 (2.0-3.1)	12.5 (10.9-14.1)		1.5 (1.1-1.9)
2 Admissions	7.2 (5.1-9.3)	2.1 (1.3-3.1)	9.2 (6.0-12.2)		0.6 (0.3-1.0)
≥3 Admissions	4.1 (2.1–6.4)	1.7 (0.7-3.1)	3.9 (1.7-6.6)		0.8 (0.3-1.7)
75					
0 Admissions	10.7 (10.2-11.2)	4.1 (3.6-4.6)	10.7 (9.8-11.5)		2.3 (1.8-2.8)
1 Admissions	7.0 (6.2-7.9)	2.4 (2.0-2.9)	9.0 (7.8-10.3)		1.5 (1.1-1.9)
2 Admissions	4.7 (3.3-6.2)	2.0 (1.2-2.9)	6.5 (3.9-9.1)		0.6 (0.3-1.1)
≥3 Admissions	2.6 (1.3-4.2)	1.7 (0.7-3.0)	2.5 (1.1-4.2)		0.8 (0.3-1.6)
80					
0 Admissions	7.3 (7.0-7.7)	4.0 (3.6-4.5)	7.4 (6.9-8.1)		2.3 (1.9-2.9)
1 Admissions	4.5 (4.0-5.1)	2.3 (1.9-2.8)	6.1 (5.2-6.9)		1.5 (1.2-1.9)
2 Admissions	2.8 (2.0-3.9)	1.9 (1.2-2.7)	4.3 (2.7-6.1)		0.6 (0.3-1.0)
≥3 Admissions	1.6 (0.9-2.7)	1.7 (0.8-2.9)	1.5 (0.7-2.6)		0.9 (0.4-1.7)
85					
0 Admissions	4.7 (4.4-5.0)	3.9 (3.4-4.4)	4.9 (4.4-5.4)		2.4 (1.9-3.2)
1 Admissions	2.7 (2.4-3.1)	2.2 (1.8-2.7)	3.8 (3.3-4.5)		1.6 (1.2-2.0)
2 Admissions	1.7 (1.2-2.3)	1.7 (1.2-2.5)	2.7 (1.8-3.8)		0.7 (0.4-1.0)
≥3 Admissions	1.0 (0.5-1.6)	1.6 (0.8-2.7)	0.9 (0.5-1.5)		0.9 (0.4-1.7)
90					
0 Admissions	2.8 (2.6-3.1)	3.7 (3.2-4.3)	3.0 (2.5-3.5)		2.5 (1.9-3.6)
1 Admissions	1.6 (1.4-1.8)	2.0 (1.6-2.5)	2.3 (1.9-2.8)		1.6 (1.2-2.2)
2 Admissions	1.0 (0.7-1.3)	1.6 (1.1-2.3)	1.6 (1.0-2.4)		0.7 (0.4-1.1)
≥3 Admissions	0.6 (0.3-1.0)	1.5 (0.7-2.6)	0.5 (0.3-0.8)		1.0 (0.5-1.8)

**Table 2.** Life Expectancy According to Age and Major Surgical Admissions by Sex

Age, y	Life expectancy, mean (95% CI), y			
	Females		Males	
	Active	Disabled	Active	Disabled
70				
0 Admissions	13.7 (12.9-14.3)	3.6 (3.2-4.1)	13.7 (12.5-14.7)	1.9 (1.6-2.4)
1 Admissions	14.1 (12.7-15.2)	3.6 (2.9-4.4)	13.9 (12.3-15.3)	1.9 (1.4-2.3)
2 Admissions	12.5 (10.8-14.0)	2.9 (2.2-3.7)	13.4 (10.8-15.9)	1.4 (0.8-2.0)
≥3 Admissions	9.2 (7.4-11.0)	3.5 (2.4-5.0)	13.9 (10.4-17.1)	1.1 (0.6-1.9)
75				
0 Admissions	9.9 (9.3-10.5)	3.6 (3.2-4.1)	9.9 (9.1-10.7)	2.0 (1.6-2.5)
1 Admissions	10.3 (9.3-11.2)	3.6 (2.9-4.3)	10.0 (8.9-11.1)	1.9 (1.5-2.4)
2 Admissions	9.0 (7.7-10.3)	2.9 (2.2-3.7)	9.8 (7.8-11.6)	1.4 (0.9-2.1)
≥3 Admissions	6.2 (4.9-7.4)	3.5 (2.4-4.8)	10.1 (7.3-12.9)	1.1 (0.6-1.8)
80				
0 Admissions	6.7 (6.3-7.2)	3.5 (3.1-4.0)	6.7 (6.1-7.3)	2.0 (1.6-2.5)
1 Admissions	7.0 (6.3-7.7)	3.5 (2.9-4.3)	6.7 (5.9-7.5)	1.9 (1.5-2.5)
2 Admissions	6.0 (5.2-6.9)	2.8 (2.1-3.6)	6.6 (5.3-8.1)	1.4 (0.9-2.0)
≥3 Admissions	3.9 (3.1-4.7)	3.4 (2.4-4.6)	6.9 (4.8-9.2)	1.1 (0.6-1.8)
85				
0 Admissions	4.3 (4.0-4.6)	3.4 (2.9-3.9)	4.2 (3.7-4.7)	2.1 (1.6-2.7)
1 Admissions	4.5 (4.0-5.0)	3.4 (2.8-4.1)	4.2 (3.7-4.8)	1.9 (1.5-2.5)
2 Admissions	3.8 (3.2-4.4)	2.7 (2.1-3.5)	4.2 (3.3-5.4)	1.4 (0.9-2.1)
≥3 Admissions	2.3 (1.8-2.8)	3.2 (2.3-4.4)	4.4 (3.0-6.0)	1.1 (0.7-1.8)
90				
0 Admissions	2.6 (2.3-2.8)	3.2 (2.7-3.9)	2.5 (2.1-2.9)	2.2 (1.6-3.0)
1 Admissions	2.7 (2.3-3.1)	3.2 (2.6-4.0)	2.4 (2.1-2.9)	1.9 (1.4-2.6)
2 Admissions	2.2 (1.9-2.6)	2.6 (1.9-3.4)	2.5 (1.9-3.2)	1.5 (1.0-2.3)
≥3 Admissions	1.3 (1.1-1.6)	3.0 (2.1-4.0)	2.7 (1.9-3.8)	1.2 (0.7-1.9)

**Table 3.** Life Expectancy According to Age and Other Hospitalization Admissions by Sex

Age, y	Life expectancy, mean (95% CI), y^a^			
	Females		Males	
	Active	Disabled	Active	Disabled
70				
0 Admissions	19.6 (17.8-21.4)	5.9 (4.2-8.5)	19.5 (17.1-21.8)	3.3 (1.9-5.5)
1 Admissions	12.6 (11.1-14.1)	3.9 (3.0-5.0)	14.6 (12.5-16.4)	2.4 (1.7-3.4)
2 Admissions	9.0 (7.5-10.5)	3.9 (2.9-5.2)	11.3 (8.9-13.4)	2.2 (1.4-3.2)
≥3 Admissions	6.2 (5.2-7.3)	2.6 (2.0-3.3)	7.2 (5.6-8.9)	1.3 (0.9-1.9)
75				
0 Admissions	15.8 (14.2-17.6)	5.9 (4.2-8.6)	15.4 (13.1-17.9)	3.2 (1.9-6.0)
1 Admissions	9.7 (8.5-10.8)	4.0 (3.0-5.1)	10.8 (9.2-12.4)	2.3 (1.6-3.2)
2 Admissions	6.5 (5.5-7.6)	3.9 (3.0-5.1)	8.0 (6.4-9.7)	2.1 (1.4-3.0)
≥3 Admissions	4.4 (3.7-5.1)	2.6 (2.1-3.2)	4.8 (3.8-5.8)	1.2 (0.9-1.6)
80				
0 Admissions	12.3 (10.9-14.1)	5.9 (4.2-8.8)	11.9 (10.0-14.1)	3.5 (2.0-6.0)
1 Admissions	7.2 (6.3-8.1)	4.1 (3.1-5.3)	8.1 (6.8-9.4)	2.6 (1.7-3.7)
2 Admissions	4.6 (3.8-5.3)	3.9 (2.9-5.1)	5.8 (4.6-7.0)	2.4 (1.5-3.4)
≥3 Admissions	3.0 (2.6-3.4)	2.6 (2.2-3.0)	3.3 (2.7-3.9)	1.5 (1.1-1.9)
85				
0 Admissions	9.3 (8.0-11.0)	6.0 (4.1-9.0)	8.8 (7.1-11.0)	3.6 (2.0-6.4)
1 Admissions	5.1 (4.4-6.0)	4.2 (3.1-5.5)	5.6 (4.6-6.7)	2.7 (1.8-4.1)
2 Admissions	3.1 (2.6-3.7)	3.9 (2.9-5.1)	3.8 (3.1-4.7)	2.5 (1.6-3.8)
≥3 Admissions	2.0 (1.8-2.2)	2.6 (2.3-3.0)	2.1 (1.8-2.5)	1.6 (1.2-2.0)
90				
0 Admissions	6.8 (5.6-8.4)	6.0 (4.0-9.4)	6.2 (4.7-8.4)	3.8 (2.0-7.0)
1 Admissions	3.6 (3.0-4.3)	4.3 (3.1-5.9)	3.7 (2.9-4.8)	2.8 (1.8-4.6)
2 Admissions	2.0 (1.7-2-5)	3.8 (2.8-5.2)	2.5 (1.9-3.1)	2.7 (1.7-4.3)
≥3 Admissions	1.4 (1.2-1.5)	2.6 (2.2-3.0)	1.4 (1.1-1.6)	1.7 (1.3-2.3)

^a^ Values are adjusted for time-varying exposure to critical illness or major surgical procedure.

## Discussion

In this prospective longitudinal cohort study of community-living older persons who were not disabled, we evaluated how estimates of active and disabled life expectancy differed based on exposure to intervening illnesses and injuries, with 3 major findings that warrant comment. First, active life expectancy decreased monotonically for each age group as the number of hospital admissions increased for critical illness, major nonelective surgical procedures, and reasons other than critical illness or major surgical procedure but not for major elective surgical procedures. Second, disabled life expectancy decreased for each age group, although not monotonically, as the number of hospital admissions increased for critical illness and reasons other than critical illness or major surgical procedure. Third, disabled life expectancy changed relatively little as the number of hospital admissions increased for all major surgical procedures, including elective and nonelective surgical procedures. These findings provide strong evidence that exposure to serious intervening illnesses and injuries but not major elective surgical procedures is associated with considerably diminished active life expectancy.

Outcomes evaluated in this study, active and disabled life expectancy, are important not only to policymakers, but also to older persons, who consistently identify the maintenance of independent function as a top health outcome priority.^1,28^ Decrements observed in active life expectancy in the setting of critical illness, major nonelective surgical procedures, and hospitalization for reasons other than critical illness or major surgical procedure were pronounced and clinically meaningful. For an individual aged 70 years, active life expectancy decreased from 14.6 years in the absence of a critical illness to 4.0 years in the setting of 3 or more critical illness admissions, from 13.9 years in the absence of a nonelective major surgical procedure to 9.2 years in the setting of 2 or more nonelective major surgical procedures, and from 19.4 years in the absence of other hospitalizations to 7.0 years in the setting of 3 or more other hospitalizations. With each type of serious intervening illness and injury, the magnitude of reductions in active life expectancy progressively decreased with advancing age, a finding that can be explained by the strong association of age with disability and death.^11^

For critical illness and other hospitalization, decrements observed in disabled life expectancy were less pronounced than those in active life expectancy. This was likely due to the much higher risk of death among older persons who become disabled.^29^ For major surgical procedure, including elective and nonelective operations, decrements were not observed in disabled life expectancy as the number of these events increased or with advancing age, leading to point estimates with a relatively narrow range. This could be attributable, at least in part, to selection effects, with elective and nonelective surgical procedures being preferentially offered to or accepted by older persons who had a lower expected mortality. Selection effects could also potentially explain why active life expectancy was consistently lower for persons who had no elective surgical procedures vs those who had 1 such surgical procedure. Additionally, many elective surgical procedures, such as hip and knee replacements, often lead to improvements in function.^30,31^

For each type of intervening event, findings for active and disabled life expectancy did not differ substantively between female and male participants. Although this was not a focus of this study, females consistently spent a greater proportion of their remaining life disabled than males, a finding that has been demonstrated repeatedly in prior studies,^6,32,33^ bolstering the validity of our estimates.

With the aging of the population, the number of older US residents with disability is expected to increase considerably over the next 2 decades.^34^ This increase will have pronounced effects on not only quality of life but also health care spending, with the cost for long-term care projected to increase from $207 billion in 2020 to $346 billion in 2040.^35^ Results of this study suggest that at least 4 types of evidence-based strategies should be prioritized by clinicians and policymakers to reduce the burden of disability and, in turn, increase active life expectancy among older persons. First, minimize preventable illnesses and injuries that lead to hospitalization for critical illness, major nonelective surgical procedures, and reasons other than critical illness or major surgical procedures, including falls, strokes, infections, and exacerbations of heart failure and chronic lung disease, among others.^36,37,38,39^ Second, decrease adverse functional consequences of these hospitalizations, for example through early mobilization, prevention of delirium, and use of acute geriatric units.^40,41,42,43,44^ Third, bolster restorative therapies after hospitalization, for example through augmented home-care services and more robust rehabilitation.^45,46^ Fourth, substitute hospital at home when possible for traditional inpatient care.^47^ In addition, enhancing caregiver engagement and education is a promising, although not well-tested strategy for facilitating functional recovery after hospitalization.^48^

Major strengths of this study include monthly assessments of disability with little missing data and the long duration of follow-up with relatively little attrition for reasons other than death. The availability of monthly data allowed us to account for high rates of transitions between states of disability and independence^29^ and should lead to estimates of active and disabled life expectancy that are more accurate than those based on less frequent assessments. In addition, the ascertainment and classification of intervening illnesses and injuries was rigorous and complete, with Medicare claims data serving as the primary source of information, complemented by careful review of medical records.

### Limitations

Our study has several limitations. First, our analysis did not adjust for factors, such as comorbidity, cognitive impairment, and social determinants of health, that may be associated with both intervening events and functional well-being. Our objective was to determine how estimates of active and disabled life expectancy differed based on exposure to intervening illnesses and injuries not to evaluate independent association of these events with functional well-being. Nonetheless, the frequency of our assessments increases the likelihood that intervening events preceded transitions between states of independence and disability, thereby strengthening temporal precedence. Second, our assessment of disability did not include higher-level activities, such as outdoor mobility or shopping, that could also be adversely affected by intervening events. Most studies of active and disabled life expectancy, including the original study by Katz et al,^3^ have focused on essential activities of daily living. Third, based on prior research,^12^ we evaluated other hospitalization using a hierarchical approach, meaning that months classified as no other hospitalization could not include a critical illness or major surgical procedure. This likely explains the higher estimates of active life expectancy across age groups for no other hospitalization than for no critical illness and no major surgical procedure, both of which could have included one of the other types of intervening events. Fourth, it was not possible to determine whether the associations were attributable to the underlying illness or injury or the resulting hospitalization. Evidence-based strategies we proposed to reduce the burden of disability address each of these mechanisms. Fifth, because our study participants were members of a single health plan in south-central Connecticut, our results may not be generalizable to older persons in other settings. The generalizability of our results is enhanced by our high participation rate, which was greater than 75%, and low rate of attrition.^49^

## Conclusions

In this cohort study, active life expectancy among community-living older persons who were not disabled was considerably diminished in the setting of serious intervening illnesses and injuries. These findings suggest that prevention and more aggressive management of these events, together with restorative interventions, have the potential to improve the functional well-being of older persons.

## Notes

Supplement 1. **eMethods.** Categorizing Reasons for Critical Illness Admissions and Other Hospitalizations and Types of Major Surgical Procedure **eTable 1.** Reasons for Critical Illness Admissions **eTable 2.** Types of Major Surgical Procedure **eTable 3.** Reasons for Other Hospitalizations **eTable 4.** Active and Disabled Life Expectancy According to Age and Number of Admissions for Critical Illness, Major Surgical Procedure, and Other Hospitalization **eTable 5.** Total Life Expectancy According to Age and Number of Admissions for Critical Illness, Major Surgical Procedure, and Other Hospitalization **eTable 6.** Active and Disabled Life Expectancy According to Age and Number of Admissions for Elective and Nonelective Major Surgical Procedure **eTable 7.** Total Life Expectancy According to Age, Sex, and Number of Admissions for Critical Illness, Major Surgical Procedure, and Other Hospitalization

Supplement 2.
Data Sharing Statement

## References

[1] Fried TR, Tinetti ME, Iannone L, O’Leary JR, Towle V, Van Ness PH. Health outcome prioritization as a tool for decision making among older persons with multiple chronic conditions. Arch Intern Med. 2011;171(20):1854-1856. doi:10.1001/archinternmed.2011.424 21949032 PMC4036681

[2] Manton KG, Stallard E, Liu K. Forecasts of active life expectancy: policy and fiscal implications. J Gerontol. 1993;48(Spec No):11-26. doi:10.1093/geronj/48.Special_Issue.11 8409235

[3] Katz S, Branch LG, Branson MH, Papsidero JA, Beck JC, Greer DS. Active life expectancy. N Engl J Med. 1983;309(20):1218-1224. doi:10.1056/NEJM198311173092005 6633571

[4] Kingston A, Wohland P, Wittenberg R, ; Cognitive Function and Ageing Studies collaboration. Is late-life dependency increasing or not: a comparison of the Cognitive Function and Ageing Studies (CFAS). Lancet. 2017;390(10103):1676-1684. doi:10.1016/S0140-6736(17)31575-1 28821408 PMC5640505

[5] Crimmins EM, Zhang Y, Saito Y. Trends over 4 decades in disability-free life expectancy in the United States. Am J Public Health. 2016;106(7):1287-1293. doi:10.2105/AJPH.2016.303120 27077352 PMC4984740

[6] Freedman VA, Wolf DA, Spillman BC. Disability-free life expectancy over 30 years: a growing female disadvantage in the US population. Am J Public Health. 2016;106(6):1079-1085. doi:10.2105/AJPH.2016.303089 26985619 PMC4860065

[7] Davies LE, Mercer SW, Brittain K, Jagger C, Robinson L, Kingston A. The association between multimorbidity and mobility disability-free life expectancy in adults aged 85 years and over: a modelling study in the Newcastle 85+ cohort. PLoS Med. 2022;19(11):e1004130. doi:10.1371/journal.pmed.1004130 36374907 PMC9662726

[8] Jagger C, Matthews R, Matthews F, Robinson T, Robine JM, Brayne C; Medical Research Council Cognitive Function and Ageing Study Investigators. The burden of diseases on disability-free life expectancy in later life. J Gerontol A Biol Sci Med Sci. 2007;62(4):408-414. doi:10.1093/gerona/62.4.408 17452735

[9] Guralnik JM, Land KC, Blazer D, Fillenbaum GG, Branch LG. Educational status and active life expectancy among older Blacks and Whites. N Engl J Med. 1993;329(2):110-116. doi:10.1056/NEJM199307083290208 8510687

[10] Ferrucci L, Izmirlian G, Leveille S, . Smoking, physical activity, and active life expectancy. Am J Epidemiol. 1999;149(7):645-653. doi:10.1093/oxfordjournals.aje.a009865 10192312

[11] Gill TM, Gahbauer EA, Murphy TE, Han L, Allore HG. Risk factors and precipitants of long-term disability in community mobility: a cohort study of older persons. Ann Intern Med. 2012;156(2):131-140. doi:10.7326/0003-4819-156-2-201201170-00009 22250144 PMC3278794

[12] Gill TM, Han L, Gahbauer EA, Leo-Summers L, Murphy TE. Risk factors and precipitants of severe disability among community-living older persons. JAMA Netw Open. 2020;3(6):e206021. doi:10.1001/jamanetworkopen.2020.6021 32484551 PMC7267844

[13] Stabenau HF, Becher RD, Gahbauer EA, Leo-Summers L, Allore HG, Gill TM. Functional trajectories before and after major surgery in older adults. Ann Surg. 2018;268(6):911-917. doi:10.1097/SLA.0000000000002659 29356710 PMC6521949

[14] Ferrante LE, Pisani MA, Murphy TE, Gahbauer EA, Leo-Summers LS, Gill TM. Functional trajectories among older persons before and after critical illness. JAMA Intern Med. 2015;175(4):523-529. doi:10.1001/jamainternmed.2014.7889 25665067 PMC4467795

[15] Gill TM, Desai MM, Gahbauer EA, Holford TR, Williams CS. Restricted activity among community-living older persons: incidence, precipitants, and health care utilization. Ann Intern Med. 2001;135(5):313-321. doi:10.7326/0003-4819-135-5-200109040-00007 11529694

[16] Gill TM, Kurland BF. Prognostic effect of prior disability episodes among nondisabled community-living older persons. Am J Epidemiol. 2003;158(11):1090-1096. doi:10.1093/aje/kwg237 14630605

[17] Gill TM, Hardy SE, Williams CS. Underestimation of disability in community-living older persons. J Am Geriatr Soc. 2002;50(9):1492-1497. doi:10.1046/j.1532-5415.2002.50403.x 12383145

[18] Gill TM, Han L, Gahbauer EA, Leo-Summers L, Murphy TE. Cohort profile: the precipitating events project (PEP study). J Nutr Health Aging. 2020;24(4):438-444. doi:10.1007/s12603-020-1341-4 32242212 PMC7322244

[19] Folstein MF, Folstein SE, McHugh PR. “Mini-mental state”: a practical method for grading the cognitive state of patients for the clinician. J Psychiatr Res. 1975;12(3):189-198. doi:10.1016/0022-3956(75)90026-6 1202204

[20] Gill TM. Disentangling the disabling process: insights from the precipitating events project. Gerontologist. 2014;54(4):533-549. doi:10.1093/geront/gnu067 25035454 PMC4155452

[21] Gill TM, Allore H, Holford TR, Guo Z. The development of insidious disability in activities of daily living among community-living older persons. Am J Med. 2004;117(7):484-491. doi:10.1016/j.amjmed.2004.05.018 15464705

[22] Schwarze ML, Barnato AE, Rathouz PJ, . Development of a list of high-risk operations for patients 65 years and older. JAMA Surg. 2015;150(4):325-331. doi:10.1001/jamasurg.2014.1819 25692282 PMC4414395

[23] Research Data Assistance Center. Inpatient admission type code. Accessed February 24, 2025. https://resdac.org/cms-data/variables/inpatient-admission-type-code

[24] Hardy SE, Gill TM. Recovery from disability among community-dwelling older persons. JAMA. 2004;291(13):1596-1602. doi:10.1001/jama.291.13.1596 15069047

[25] Gill TM, Guo Z, Allore HG. Subtypes of disability in older persons over the course of nearly 8 years. J Am Geriatr Soc. 2008;56(3):436-443. doi:10.1111/j.1532-5415.2007.01603.x 18194225 PMC2799685

[26] Lee M, Rendall MS. Self-employment disadvantage in the working lives of Blacks and females. Popul Res Policy Rev. 2001;20:291-320. doi:10.1023/A:1011887013195

[27] Efron B, Tibshirani RJ. An Introduction to the Bootstrap. Chapman and Hall; 1994. doi:10.1201/9780429246593

[28] Davenport C, Ouellet J, Tinetti ME. Use of the patient-identified top health priority in care decision-making for older adults with multiple chronic conditions. JAMA Netw Open. 2021;4(10):e2131496. doi:10.1001/jamanetworkopen.2021.31496 34709390 PMC8554637

[29] Hardy SE, Dubin JA, Holford TR, Gill TM. Transitions between states of disability and independence among older persons. Am J Epidemiol. 2005;161(6):575-584. doi:10.1093/aje/kwi083 15746474

[30] Price AJ, Alvand A, Troelsen A, . Knee replacement. Lancet. 2018;392(10158):1672-1682. doi:10.1016/S0140-6736(18)32344-4 30496082

[31] Ferguson RJ, Palmer AJ, Taylor A, Porter ML, Malchau H, Glyn-Jones S. Hip replacement. Lancet. 2018;392(10158):1662-1671. doi:10.1016/S0140-6736(18)31777-X 30496081

[32] Chatterji S, Byles J, Cutler D, Seeman T, Verdes E. Health, functioning, and disability in older adults–present status and future implications. Lancet. 2015;385(9967):563-575. doi:10.1016/S0140-6736(14)61462-8 25468158 PMC4882096

[33] Fried LP, Guralnik JM. Disability in older adults: evidence regarding significance, etiology, and risk. J Am Geriatr Soc. 1997;45(1):92-100. doi:10.1111/j.1532-5415.1997.tb00986.x 8994496

[34] Administration on Aging, Administration for Community Living, US Department of Health and Human Services. 2023 Profile of older Americans. Accessed February 24, 2025. https://acl.gov/sites/default/files/Profile%20of%20OA/ACL_ProfileOlderAmericans2023_508.pdf

[35] Stevenson DG. Planning for the future—long-term care and the 2008 election. N Engl J Med. 2008;358(19):1985-1987. doi:10.1056/NEJMp0802347 18463373

[36] Tinetti ME. Clinical practice: preventing falls in elderly persons. N Engl J Med. 2003;348(1):42-49. doi:10.1056/NEJMcp020719 12510042

[37] Straus SE, Majumdar SR, McAlister FA. New evidence for stroke prevention: clinical applications. JAMA. 2002;288(11):1396-1398. doi:10.1001/jama.288.11.1396 12234234

[38] Kim DK, Hunter P; Advisory Committee on Immunization Practices. Recommended adult immunization schedule, United States, 2019. Ann Intern Med. 2019;170(3):182-192. doi:10.7326/M18-3600 30716757

[39] Kastner M, Cardoso R, Lai Y, . Effectiveness of interventions for managing multiple high-burden chronic diseases in older adults: a systematic review and meta-analysis. CMAJ. 2018;190(34):E1004-E1012. doi:10.1503/cmaj.171391 30150242 PMC6110649

[40] Rich MW. Heart failure in the 21st century: a cardiogeriatric syndrome. J Gerontol A Biol Sci Med Sci. 2001;56(2):M88-M96. doi:10.1093/gerona/56.2.M88 11213282

[41] Landefeld CS, Palmer RM, Kresevic DM, Fortinsky RH, Kowal J. A randomized trial of care in a hospital medical unit especially designed to improve the functional outcomes of acutely ill older patients. N Engl J Med. 1995;332(20):1338-1344. doi:10.1056/NEJM199505183322006 7715644

[42] Cohen HJ, Feussner JR, Weinberger M, . A controlled trial of inpatient and outpatient geriatric evaluation and management. N Engl J Med. 2002;346(12):905-912. doi:10.1056/NEJMsa010285 11907291

[43] Inouye SK. Delirium in older persons. N Engl J Med. 2006;354(11):1157-1165. doi:10.1056/NEJMra052321 16540616

[44] Detsky AS, Krumholz HM. Reducing the trauma of hospitalization. JAMA. 2014;311(21):2169-2170. doi:10.1001/jama.2014.3695 24788549

[45] Tinetti ME, Baker D, Gallo WT, Nanda A, Charpentier P, O’Leary J. Evaluation of restorative care vs usual care for older adults receiving an acute episode of home care. JAMA. 2002;287(16):2098-2105. doi:10.1001/jama.287.16.2098 11966384

[46] Hoenig H, Nusbaum N, Brummel-Smith K. Geriatric rehabilitation: state of the art. J Am Geriatr Soc. 1997;45(11):1371-1381. doi:10.1111/j.1532-5415.1997.tb02939.x 9361665

[47] Federman AD, Soones T, DeCherrie LV, Leff B, Siu AL. Association of a bundled hospital-at-home and 30-day postacute transitional care program with clinical outcomes and patient experiences. JAMA Intern Med. 2018;178(8):1033-1040. doi:10.1001/jamainternmed.2018.2562 29946693 PMC6143103

[48] Liebzeit D, Rutkowski R, Arbaje AI, Fields B, Werner NE. A scoping review of interventions for older adults transitioning from hospital to home. J Am Geriatr Soc. 2021;69(10):2950-2962. doi:10.1111/jgs.17323 34145906 PMC8497409

[49] Szklo M. Population-based cohort studies. Epidemiol Rev. 1998;20(1):81-90. doi:10.1093/oxfordjournals.epirev.a017974 9762511

