# Supplementary material for: Critical Illness, Major Surgery, and Other Hospitalizations and Active and Disabled Life Expectancy
Source: JAMA Netw Open. 2025 Apr 3;8(4):e254208. doi: 10.1001/jamanetworkopen.2025.4208 (PMC11969285; doi:10.1001/jamanetworkopen.2025.4208)
Supplement: Supplement 1. — eMethods. Categorizing Reasons for Critical Illness Admissions and Other Hospitalizations and Types of Major Surgical Procedure eTable 1. Reasons for Critical Illness Admissions eTable 2. Types of Major Surgical Procedure eTable 3. Reasons for Other Hospitalizations eTable 4. Active and Disabled Life Expectancy According to Age and Number of Admissions for Critical Illness, Major Surgical Procedure, and Other Hospitalization eTable 5. Total Life Expectancy According to Age and Number of Admissions for Critical Illness, Major Surgical Procedure, and Other Hospitalization eTable 6. Active and Disabled Life Expectancy According to Age and Number of Admissions for Elective and Nonelective Major Surgical Procedure eTable 7. Total Life Expectancy According to Age, Sex, and Number of Admissions for Critical Illness, Major Surgical Procedure, and Other Hospitalization [file jamanetwopen-e254208-s001.pdf]

Supplemental Online Content

Gill TM, Zang EX, Leo-Summers L, et al. Critical illness, major surgery, and other hospitalizations and active and disabled life expectancy. *JAMA Netw Open*. 2025;8(4):e254208. doi:10.1001/jamanetworkopen.2025.4208

eMethods. Categorizing Reasons for Critical Illness Admissions and Other Hospitalizations and Types of Major Surgical Procedure

eTable 1. Reasons for Critical Illness Admissions

eTable 2. Types of Major Surgical Procedure

eTable 3. Reasons for Other Hospitalizations

eTable 4. Active and Disabled Life Expectancy According to Age and Number of Admissions for Critical Illness, Major Surgical Procedure, and Other Hospitalization

eTable 5. Total Life Expectancy According to Age and Number of Admissions for Critical Illness, Major Surgical Procedure, and Other Hospitalization

eTable 6. Active and Disabled Life Expectancy According to Age and Number of Admissions for Elective and Nonelective Major Surgical Procedure

eTable 7. Total Life Expectancy According to Age, Sex, and Number of Admissions for Critical Illness, Major Surgical Procedure, and Other Hospitalization

This supplemental material has been provided by the authors to give readers additional information about their work.

© 2025 Gill TM et al. *JAMA Netw Open*.

eMethods. Categorizing Reasons for Critical Illness Admissions and Other Hospitalizations and Types of Major Surgical Procedure

The reasons for critical illness admissions and other hospitalizations were grouped into distinct diagnostic categories using a revised version of the protocol described by Ferrucci et al.[1] This protocol included the following diagnoses: cancer, diabetes, dehydration, coronary heart disease, peripheral arteriopathy, pneumonia, chronic obstructive pulmonary disease, gastrointestinal bleeding, hip fracture, congestive heart failure, arthritis or osteoporosis, and dementia. Because our cohort was considerably smaller than that analyzed by Ferrucci et al. (754 vs. 6070), we chose to group critical illness admissions and other hospitalizations into their most common diagnostic categories, respectively. For critical illness, these categories included: cardiac, infection, respiratory, gastrointestinal, neurologic, cancer, musculoskeletal and trauma, vascular, metabolic, renal and immunologic, other injury and poisoning, and other. For other hospitalizations, these categories included: infection, cardiac, respiratory, stroke, dementia and other psychiatric, fall-related injury, gastrointestinal bleeding, dehydration, cancer, musculoskeletal, other medical, and minor surgery. As described in an earlier report,[2] we categorized each major surgery into the most common types, which included: musculoskeletal, abdominal, vascular (for endovascular surgeries, noncoronary bypass grafts, and amputations), cardiothoracic, neurologic (for brain and spine surgeries), and other (including thyroidectomies, major breast operations, extensive lymph node excisions, burn debridements, and skin grafts).

1. Ferrante LE, Murphy TE, Leo-Summers LS, Gahbauer EA, Pisani MA, Gill TM. The combined effects of frailty and cognitive impairment on post-ICU disability among older ICU survivors. *Am J Respir Crit Care Med.* 2019;200:107-110.

2. Stabenau HF, Becher RD, Gahbauer EA, Leo-Summers L, Allore HG, Gill TM. Functional trajectories before and after major surgery in older adults. *Ann Surg.* 2018;268:911-917.

© 2025 Gill TM et al. *JAMA Netw Open*.

eTable 1. Reasons for Critical Illness Admissions (N=471) *

	n (%)
Cardiac	153 (32.8)
Infection	85 (18.0)
Respiratory	44 (9.3)
Gastrointestinal	41 (8.7)
Neurologic	39 (8.3)
Cancer	31 (6.6)
Musculoskeletal and trauma	27 (5.7)
Vascular	19 (4.1)
Metabolic, renal and immunologic	15 (3.2)
Other injury and poisoning	9 (1.9)
Other	8 (1.7)

* These admissions were identified from 313 participants. The reasons are presented in order of highest to lowest rates.

© 2025 Gill TM et al. *JAMA Netw Open*.

eTable 2. Types of Major Surgical Procedure *

	All Major Surgeries (N=616) †	Elective Surgical Procedure (N=396)	Non-elective Surgical Procedure (N=220)
	n (%)	n (%)	n (%)
Musculoskeletal	233 (37.8)	131 (33.1)	102 (46.4)
Abdominal	111 (18.0)	63 (15.9)	48 (21.8)
Vascular	101 (16.4)	74 (18.7)	27 (12.3)
Cardiothoracic	44 (7.1)	30 (7.6)	14 (6.4)
Neurologic	27 (4.4)	22 (5.6)	5 (2.3)
Other ‡	100 (16.2)	76 (19.2)	24 (10.9)

* The first 5 types are presented in order of highest to lowest rates for all major surgeries.

† These surgeries were identified from 352 participants: 220 for elective and 166 for non-elective.

‡ Includes thyroidectomies, major breast operations, extensive lymph node dissections, burn debridements, and skin grafts.

© 2025 Gill TM et al. *JAMA Netw Open*.

eTable 3. Reasons for Other Hospitalizations (N=2642) *

	n (%)
Infection	608 (23.0)
Cardiac	529 (20.0)
Respiratory	138 (5.2)
Stroke	123 (4.7)
Dementia and other psychiatric	107 (4.1)
Fall-related injury	98 (3.7)
Gastrointestinal bleeding	81 (3.1)
Dehydration	78 (3.0)
Cancer	63 (2.4)
Musculoskeletal	60 (2.3)
Other medical	709 (26.8)
Minor surgery	48 (1.8)

* These admissions were identified from 646 participants. The reasons are presented in order of highest to lowest rates except for Other medical.

© 2025 Gill TM et al. *JAMA Netw Open*.

eTable 4. Active and Disabled Life Expectancy According to Age and Number of Admissions for Critical Illness, Major Surgical Procedure and Other Hospitalization

		Critical Illness *		Major Surgical Procedure *		Other Hospitalization †	
Age, y	Number of Admissions	Active Life Expectancy, y	Disabled Life Expectancy, y	Active Life Expectancy, y	Disabled Life Expectancy, y	Active Life Expectancy, y	Disabled Life Expectancy, y
		Mean (95% CI)		Mean (95% CI)		Mean (95% CI)	
70	0	14.6 (13.9-15.2)	3.4 (3.0-3.7)	13.7 (13.1-14.3)	3.0 (2.7-3.3)	19.4 (18.0-20.8)	4.4 (3.5-5.8)
	1	11.3 (10.3-12.2)	2.2 (1.8-2.5)	14.0 (13.0-14.9)	2.9 (2.4-3.4)	13.5 (12.2-14.7)	3.4 (2.8-4.1)
	2	8.1 (6.3-9.9)	1.4 (1.0-1.9)	12.8 (11.3-14.1)	2.3 (1.8-2.9)	10.0 (8.9-11.2)	3.4 (2.7-4.2)
	3 or more	4.0 (2.6-5.7)	1.4 (0.8-2.3)	10.6 (8.9-12.2)	2.7 (1.9-3.5)	7.0 (6.1-7.9)	2.3 (1.9-2.8)
75	0	10.7 (10.3-11.2)	3.4 (3.0-3.7)	9.9 (9.5-10.4)	2.9 (2.6-3.3)	15.5 (14.3-16.9)	4.5 (3.4-5.9)
	1	7.9 (7.1-8.6)	2.1 (1.8-2.5)	10.2 (9.4-10.9)	2.8 (2.4-3.3)	10.3 (9.3-11.2)	3.4 (2.8-4.2)
	2	5.4 (4.2-6.7)	1.4 (1.0-1.9)	9.3 (8.2-10.3)	2.3 (1.8-2.9)	7.3 (6.5-8.1)	3.4 (2.7-4.2)
	3 or more	2.5 (1.6-3.6)	1.4 (0.8-2.2)	7.4 (6.2-8.5)	2.7 (2.0-3.5)	4.9 (4.4-5.5)	2.3 (2.0-2.7)
80	0	7.4 (7.1-7.8)	3.3 (3.0-3.7)	6.7 (6.4-7.1)	2.9 (2.6-3.2)	12.0 (10.9-13.3)	4.5 (3.4-6.1)
	1	5.1 (4.7-5.6)	2.1 (1.8-2.4)	6.9 (6.4-7.5)	2.8 (2.4-3.2)	7.6 (6.8-8.3)	3.5 (2.8-4.2)
	2	3.4 (2.6-4.2)	1.4 (1.0-1.8)	6.2 (5.5-7.0)	2.3 (1.8-2.8)	5.1 (4.5-5.7)	3.4 (2.7-4.1)
	3 or more	1.5 (1.0-2.1)	1.4 (0.9-2.1)	4.8 (4.0-5.5)	2.6 (2.0-3.4)	3.3 (3.0-3.7)	2.3 (2.0-2.6)
85	0	4.8 (4.5-5.1)	3.3 (2.9-3.7)	4.3 (4.0-4.6)	2.8 (2.5-3.2)	8.9 (7.9-10.2)	4.5 (3.3-6.2)
	1	3.2 (2.8-3.5)	2.0 (1.7-2.3)	4.4 (4.0-4.8)	2.7 (2.3-3.2)	5.4 (4.8-5.9)	3.5 (2.8-4.4)
	2	2.0 (1.6-2.6)	1.3 (0.9-1.7)	3.9 (3.4-4.5)	2.2 (1.8-2.7)	3.4 (3.0-3.9)	3.4 (2.7-4.2)
	3 or more	0.9 (0.6-1.3)	1.4 (0.9-2.1)	2.9 (2.4-3.5)	2.5 (1.9-3.3)	2.2 (2.0-2.4)	2.3 (2.0-2.6)
90	0	2.9 (2.7-3.1)	3.2 (2.7-3.7)	2.6 (2.4-2.8)	2.7 (2.4-3.2)	6.4 (5.5-7.5)	4.5 (3.3-6.3)
	1	1.9 (1.6-2.1)	1.9 (1.6-2.2)	2.6 (2.3-2.9)	2.6 (2.2-3.1)	3.6 (3.2-4.2)	3.6 (2.8-4.5)
	2	1.2 (0.9-1.5)	1.3 (0.9-1.6)	2.3 (2.0-2.7)	2.2 (1.7-2.8)	2.2 (1.9-2.6)	3.4 (2.7-4.2)
	3 or more	0.5 (0.4-0.7)	1.3 (0.8-2.0)	1.7 (1.4-2.0)	2.4 (1.8-3.2)	1.4 (1.3 1.6)	2.3 (2.0-2.6)

Abbreviation: y, years; CI, confidence interval.

* Values are adjusted for sex.

† As described in the Methods, values are adjusted for sex and for time-varying exposure to critical illness or major surgery.

© 2025 Gill TM et al. *JAMA Netw Open*.

eTable 5. Total Life Expectancy According to Age and Number of Admissions for Critical Illness, Major Surgical Procedure and Other Hospitalization

Age, y	Number of Admissions	Critical Illness *	Major Surgical Procedure *	Other Hospitalization †
		Mean Years (95% CI)		
70	0	17.9 (17.0-18.8)	16.6 (15.8-17.5)	24.2 (21.8-26.9)
	1	13.4 (12.2-14.6)	16.9 (15.5-18.1)	16.4 (14.7-18.2)
	2	9.5 (7.4-11.7)	15.1 (13.2-16.9)	12.9 (11.1-14.8)
	3 or more	5.5 (3.4-7.7)	13.3 (11.1-15.5)	8.5 (7.3-9.8)
75	0	14.1 (13.4-14.9)	12.8 (12.1-13.6)	20.4 (18.1-23.2)
	1	10.0 (9.0-11.0)	13.0 (11.9-14.0)	13.4 (12.0-14.9)
	2	6.8 (5.3-8.3)	11.6 (10.0-13.0)	10.3 (8.9-11.9)
	3 or more	3.9 (2.5-5.5)	10.0 (8.3-11.8)	6.6 (5.8-7.4)
80	0	10.7 (10.1-11.3)	9.6 (9.0-10.2)	17.0 (14.7-19.7)
	1	7.2 (6.5-7.9)	9.7 (8.8-10.6)	10.8 (9.7-12.3)
	2	4.8 (3.7-5.8)	8.5 (7.5-9.7)	8.2 (7.1-9.6)
	3 or more	2.9 (1.9-4.0)	7.4 (6.0-8.8)	5.1 (4.6-5.7)
85	0	8.1 (7.4-8.7)	3.3 (2.9-3.7)	14.0 (11.7-16.8)
	1	5.2 (4.6-5.9)	7.1 (6.4-7.9)	8.7 (7.7-10.2)
	2	3.3 (2.6-4.2)	6.1 (5.3-7.1)	6.7 (5.7-7.9)
	3 or more	2.3 (1.5-3.2)	5.4 (4.4-6.5)	4.1 (3.8-4.5)
90	0	6.1 (5.4-6.7)	5.3 (4.8-5.9)	11.6 (9.3-14.5)
	1	3.8 (3.3-4.3)	5.2 (4.5-5.9)	7.2 (6.1-8.7)
	2	2.4 (1.9-3.1)	4.5 (3.8-5.4)	5.6 (4.7-6.8)
	3 or more	1.9 (1.2-2.7)	4.1 (3.3-5.1)	3.5 (3.1-3.8)

Abbreviation: y, years; CI, confidence interval.

* Values are adjusted for sex.

† As described in the Methods, values are adjusted for sex and for time-varying exposure to critical illness or major surgery.

© 2025 Gill TM et al. *JAMA Netw Open*.

eTable 6. Active and Disabled Life Expectancy According to Age and Number of Admissions for Elective and Non-Elective Major Surgical Procedure *

		Elective		Non-Elective	
Age, y	Number of Admissions †	Active Life Expectancy, y	Disabled Life Expectancy, y	Active Life Expectancy, y	Disabled Life Expectancy, y
		Mean (95% CI)		Mean (95% CI)	
70	0	13.4 (12.8-14.1)	2.9 (2.6-3.2)	13.9 13.3-14.5)	2.9 (2.6-3.1)
	1	14.6 (13.5-15.5)	2.6 (2.2-3.0)	11.7 (10.5-12.8)	2.8 (2.3-3.4)
	2 or more	12.6 (11.5-13.8)	2.9 (2.3-3.5)	9.2 (7.4-11.0)	2.8 (1.8-4.0)
75	0	9.7 (9.3-10.1)	2.9 (2.6-3.2)	10.1 (9.7-10.5)	2.8 (2.6-3.1)
	1	10.7 (9.8-11.5)	2.5 (2.2-2.9)	8.2 (7.3-9.1)	2.7 (2.3-3.3)
	2 or more	9.0 (8.1-9.9)	2.8 (2.3-3.5)	6.2 (4.9-7.7)	2.8 (1.8-4.0)
80	0	6.5 (6.2-6.8)	2.9 (2.6-3.1)	6.9 (6.6-7.2)	2.8 (2.5-3.1)
	1	7.3 (6.7-7.9)	2.5 (2.1-2.9)	5.4 (4.8-6.0)	2.7 (2.2-3.2)
	2 or more	6.0 (5.3-6.7)	2.8 (2.3-3.4)	4.0 (3.1-5.0)	2.8 (1.9-3.9)
85	0	6.5 (6.2-6.8)	2.9 (2.6-3.1)	4.4 (4.2-4.7)	2.7 (2.4-3.0)
	1	7.3 (6.7-7.9)	2.5 (2.1-2.9)	3.3 (2.9-3.7)	2.5 (2.1-3.0)
	2 or more	6.0 (5.3-6.7)	2.8 (2.3-3.4)	2.4 (1.8-3.1)	2.7 (1.9-3.8)
90	0	2.4 (2.2-2.6)	2.7 (2.3-3.1)	2.7 (2.5-2.9)	2.6 (2.3-3.0)
	1	2.8 (2.5-3.1)	2.3 (1.9-2.7)	1.9 (1.7-2.2)	2.4 (2.0-2.9)
	2 or more	2.2 (1.8-2.5)	2.6 (2.1-3.3)	1.4 (1.1-1.8)	2.7 (1.9-3.7)

Abbreviation: y, years; CI, confidence interval.

* Values are adjusted for sex.

† As described in the Methods, the exposure had to be categorized into three groups because the models with four groups did not converge due to small cell sizes for 3 or more admissions. Overall, the exposure rates (95% CI) per 1000 person-months for elective and non-elective major surgery were 4.6 (4.1-5.1) and 2.5 (2.2-3.0), respectively.

© 2025 Gill TM et al. *JAMA Netw Open*.

eTable 7. Total Life Expectancy According to Age, Sex and Number of Admissions for Critical Illness, Major Surgical Procedure and Other Hospitalization

		Critical Illness		Major Surgical Procedure		Other Hospitalization *	
Age, y	Number of Admissions	Women	Men	Women	Men	Women	Men
		Mean Years (95% CI)		Mean Years (95% CI)		Mean Years (95% CI)	
70	0	18.7 (17.5-19.7)	16.7 (15.3-18.1)	17.3 (16.1-18.3)	15.6 (14.2-16.9)	25.4 (22.2-29.6)	22.8 (19.3-27.1)
	1	12.8 (11.0-14.4)	14.0 (12.0-15.9)	17.7 (15.7-19.5)	15.8 (13.9-17.6)	16.5 (14.0-18.8)	17.0 (14.3-19.6)
	2	9.3 (6.5-11.9)	9.8 (6.4-13.1)	15.4 (13.2-17.6)	14.8 (11.7-17.7)	13.0 (10.6-15.5)	13.5 (10.3-16.4)
	3 or more	5.8 (2.8-9.0)	4.8 (2.0-8.2)	12.7 (10.0-15.6)	15.0 (11.1-18.7)	8.8 (7.2-10.5)	8.5 (6.5-10.6)
75	0	14.8 (13.9-15.6)	13.0 (11.8-14.2)	13.5 (12.6-14.4)	11.9 (10.8-13.0)	21.7 (18.6-26.1)	18.6 (15.1-23.6)
	1	9.4 (8.2-10.7)	10.4 (9.0-12.1)	13.8 (12.3-15.3)	11.9 (10.3-13.4)	13.7 (11.6-15.6)	13.1 (10.9-15.3)
	2	6.7 (4.7-8.7)	7.1 (4.3-9.9)	11.8 (10.0-13.7)	11.2 (8.8-13.5)	10.5 (8.6-12.5)	10.1 (7.9-12.6)
	3 or more	4.3 (2.1-6.6)	3.3 (1.6-5.6)	9.6 (7.5-11.9)	11.2 (8.0-14.4)	7.0 (5.9-8.1)	6.0 (4.8-7.2)
80	0	11.4 (10.6-12.2)	9.8 (8.9-10.9)	10.2 (9.4-11.1)	8.7 (7.8-9.6)	18.2 (15.2-22.7)	15.4 (12.2-19.9)
	1	6.8 (6.0-7.7)	7.6 (6.5-8.7)	10.5 (9.3-11.8)	8.6 (7.5-9.9)	11.3 (9.5-13.3)	10.6 (8.6-12.8)
	2	4.7 (3.4-6.3)	5.0 (3.1-7.0)	8.8 (7.4-10.3)	8.0 (6.3-10.0)	8.5 (6.8-10.2)	8.1 (6.2-10.4)
	3 or more	3.2 (1.8-5.0)	2.3 (1.2-4.1)	7.2 (5.7-9.0)	8.0 (5.6-10.8)	5.6 (4.9-6.3)	4.8 (4.0-5.7)
85	0	8.6 (7.9-9.4)	7.3 (6.3-8.6)	7.6 (6.9-8.5)	6.3 (5.4-7.2)	15.3 (12.2-19.7)	12.4 (9.2-17.3)
	1	4.9 (4.2-5.6)	5.4 (4.6-6.3)	7.8 (6.8-9.0)	6.1 (5.2-7.2)	9.3 (7.6-11.4)	8.3 (6.4-10.5)
	2	3.4 (2.5-4.5)	3.3 (2.2-4.7)	6.5 (5.3-7.7)	5.7 (4.3-7.4)	7.0 (5.5-8.6)	6.4 (4.8-8.3)
	3 or more	2.6 (1.5-3.9)	1.8 (1.0-3.0)	5.5 (4.2-7.0)	5.6 (3.8-7.6)	4.6 (4.1-5.1)	3.7 (3.1-4.5)
90	0	6.5 (5.8-7.4)	5.5 (4.5-6.9)	5.8 (5.1-6.7)	4.7 (3.8-5.9)	12.7 (9.7-11.5)	10.0 (6.7-15.0)
	1	3.6 (3.0-4.2)	3.9 (3.2-4.9)	5.9 (5.0-7.0)	4.4 (3.5-5.4)	7.8 (6.1-10.1)	6.6 (4.8-9.3)
	2	2.5 (1.8-3.5)	2.3 (1.5-3.3)	4.8 (3.8-5.9)	4.1 (3.0-5.4)	5.9 (4.5-7.5)	5.2 (3.7-7.3)
	3 or more	2.1 (1.1-3.3)	1.5 (0.8-2.5)	4.3 (3.2-5.5)	3.9 (2.7-5.5)	3.9 (3.5 4.4)	3.1 (2.5-3.9)

Abbreviation: y, years; CI, confidence interval.

* As described in the Methods, values are adjusted for time-varying exposure to critical illness or major surgery.

© 2025 Gill TM et al. *JAMA Netw Open*.
