# Supplementary material for: Critical Illness, Major Surgery, and Other Hospitalizations and Active and Disabled Life Expectancy
Source: JAMA Netw Open. 2025 Apr 3;8(4):e254208. doi: 10.1001/jamanetworkopen.2025.4208 (PMC11969285; doi:10.1001/jamanetworkopen.2025.4208)
Supplement: Supplement 2. — Data Sharing Statement [file jamanetwopen-e254208-s002.pdf]

Data Sharing Statement

Gill. Critical Illness, Major Surgery, and Other Hospitalizations and Active and Disabled Life Expectancy. *JAMA Netw Open*. Published online April 3, 2025. doi:10.1001/jamanetworkopen.2025.4208

Data
Data available: Yes
Data types: Deidentified participant data
How to access data: evelyne.gahbauer@yale.edu
When available: With publication

Supporting Documents
Document types: Statistical/analytic code
How to access documents: ling.han@yale.edu
When available: With publication

Additional Information
Who can access the data: researchers whose proposed use of the data has been approved
Types of analyses: for a specified purpose
Mechanisms of data availability: with investigator support, after approval of a proposal, and with a signed data access agreement
